# Protocol for the generation of *HLF+ HOXA+* human hematopoietic progenitor cells from pluripotent stem cells

**DOI:** 10.1016/j.xpro.2024.103592

**Published:** 2025-01-24

**Authors:** Sherry Li Zheng, Jonas L. Fowler, Julie Y. Chen, Christopher Li, Elaine Lin, Alana T. Nguyen, Angela Chen, George Q. Daley, Lay Teng Ang, Kyle M. Loh

**Affiliations:** 1 Institute for Stem Cell Biology & Regenerative Medicine, Stanford University, Stanford, CA 94305, USA; 2 Department of Developmental Biology, Stanford University, Stanford, CA 94305, USA; 3 Stem Cell Program, Boston Children’s Hospital, Boston, MA 02115, USA; 4 Division of Hematology/Oncology, Boston Children’s Hospital and Dana Farber Cancer Institute, Boston, MA 02115, USA; 5 Department of Biological Chemistry and Molecular Pharmacology, Harvard Medical School, Boston, MA 02115, USA; 6 Belfer Center for Science and International Affairs, Harvard Kennedy School, Cambridge, MA 02138, USA; 7 Department of Urology, Stanford University, Stanford, CA 94305, USA

**Keywords:** cell differentiation, developmental biology, stem cells

## Abstract

Hematopoietic stem cells (HSCs) generate blood and immune cells. Here, we present a protocol to differentiate human pluripotent stem cells (hPSCs) into hematopoietic progenitors that express the signature HSC transcription factors *HLF*, *HOXA5*, *HOXA7*, *HOXA9*, and *HOXA10.* hPSCs are dissociated, seeded, and then sequentially differentiated into posterior primitive streak, lateral mesoderm, artery endothelium, hemogenic endothelium, and hematopoietic progenitors through the sequential addition of defined, serum-free media. This 10-day protocol enables the manufacturing of blood and immune cells in monolayer cultures.

For complete details on the use and execution of this protocol, please refer to Fowler and Zheng et al.^1^

## Highlights

• Instructions to reconstitute differentiation factors and prepare differentiation media
• Guidance on culturing hPSCs and seeding them for differentiation
• Steps to sequentially convert hPSCs into *HLF+ HOXA+* hematopoietic progenitors

## Abstract (editor highlights)

Publisher’s note: Undertaking any experimental protocol requires adherence to local institutional guidelines for laboratory safety and ethics.

## Graphical abstract

**Figure:**
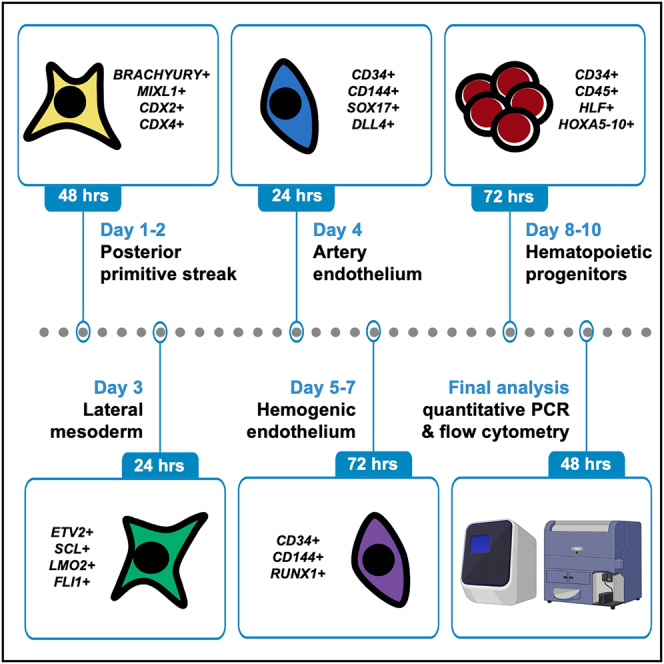

## Before you begin

Here, we describe a step-by-step method to rapidly and efficiently differentiate human pluripotent stem cells (hPSCs, including embryonic [hESCs] and induced pluripotent stem cells [hiPSCs]) sequentially into 1) posterior primitive streak, 2) lateral mesoderm, 3) arterial endothelium, 4) hemogenic endothelium, and ultimately into 5) CD34+ CD45+ hematopoietic progenitors that express *HLF* and *HOXA5-HOXA10* (referring to *HOXA5*, *HOXA7*, *HOXA9*, and *HOXA10*).^1^ This method was inspired by pioneering methods that successfully differentiated hPSCs into CD34+ hematopoietic progenitors.^2^^,^^3^^,^^4^^,^^5^^,^^6^^,^^7^^,^^8^^,^^9^^,^^10^^,^^11^^,^^12^^,^^13^^,^^14^^,^^15^^,^^16^^,^^17^^,^^18^^,^^19^^,^^20^^,^^21^^,^^22^^,^^23^^,^^24^^,^^25^ However, these methods often yielded hematopoietic progenitors that minimally expressed *HLF* and *HOXA5-HOXA10*,^10^^,^^11^^,^^12^^,^^13^^,^^14^ transcription factors that are critical to HSC identity.^12^^,^^26^^,^^27^^,^^28^^,^^29^^,^^30^^,^^31^^,^^32^^,^^33^^,^^34^^,^^35^^,^^36^ We differentiate hPSCs into *HLF*+ *HOXA5-HOXA10*+ hematopoietic progenitors in monolayer culture by sequentially adding media containing activators and inhibitors of various extracellular signaling pathways. Cells are differentiated in serum-free media, without feeder cells or genetic manipulations.

This protocol was developed using H1 and H7 hPSCs^37^ cultured in mTeSR Plus media (STEMCELL Technologies, 100–0276) on Geltrex-coated plates. It has also been successfully used to differentiate 1157 hiPSCs^16^ cultured in mTeSR Plus on Geltrex-coated plates and WTC11 hiPSCs^38^ cultured in Essential 8 (E8) media^39^ on Geltrex-coated plates. Detailed methods to culture hPSCs prior to differentiation have been described elsewhere.^40^ We recommend following those methods – and in particular, adapting undifferentiated hPSCs to growth in mTeSR Plus media and Geltrex – before attempting the differentiation protocol described here.

All differentiation steps should be performed using aseptic technique in a sterile cell culture hood.

### Institutional permissions

At Stanford University, hPSC differentiation experiments require regulatory approval from the Stem Cell Research Oversight (SCRO) committee. Readers carrying out this experimental protocol must likewise secure permission from their respective institutions.

### Reconstitute stocks of growth factors and small molecules

**Timing: 3 h**

This section describes how to reconstitute growth factors and small molecules in appropriate solvents for long-term storage prior to their use in differentiation.

**Table:** 50 mL of PBS + 0.1% BSA

Reagent	Final concentration	Amount
**PBS**	1×	50 mL
**BSA Fraction V, 7.5% solution** (Thermo Fisher, 15260-037)	0.1%	666.67 μL

Storage: PBS + 0.1% BSA can be stored at 4°C for 1 year.

1. Make PBS + 0.1% BSA by adding 666.67 μL of 7.5% BSA Fraction V (Thermo Fisher, 15260-037) to 50 mL of PBS. Sterilely filter this solution through a 0.22 μm filter unit.
2. Reconstitute recombinant growth factor proteins.
  ***Note:*** Most growth factors are soluble in PBS + 0.1% BSA and our labs have achieved best results when using PBS + 0.1% BSA over other solvents; however, manufacturer’s notes for lot-specific solubility should always be consulted before reconstituting growth factors.
  a. Reconstitute Activin A (R&D Systems, 338-AC) at 50 μg/mL using PBS + 0.1% BSA. Activin A triggers SMAD2/3 phosphorylation and TGF-β pathway activation.
  b. Reconstitute BMP4 (R&D Systems, 314-BP) at 50 μg/mL using PBS + 0.1% BSA. BMP4 is a BMP pathway activator.
  c. Reconstitute FGF2 (R&D Systems, 233-FB) at 100 μg/mL using PBS + 0.1% BSA. FGF2 is an FGF pathway activator.
  d. Reconstitute LIF (R&D Systems, 7734-LF) at 100 μg/mL using PBS + 0.1% BSA. LIF is a GP130 pathway activator.
  e. Reconstitute OSM (R&D Systems, 295-OM) at 100 μg/mL using PBS + 0.1% BSA. OSM is a GP130 pathway activator.
  f. Reconstitute VEGF-A (R&D Systems, 293-VE) at 100 μg/mL using PBS + 0.1% BSA. VEGF-A is a VEGF pathway activator.
  g. Aliquot growth factors into 1.5 mL microcentrifuge tubes.
    ***Note:*** We recommend reconstituting growth factors at high concentrations (50–100 μg/mL) because it improves protein stability. Aliquots should be prepared with reasonable working volumes, as repeated freeze/thaw cycles are detrimental to growth factor stability and should be avoided.
3. Reconstitute small molecule chemical compounds.
  ***Note:*** Most small molecules are soluble in DMSO and our labs have achieved best results when using DMSO over other solvents; however, manufacturer’s notes for lot-specific solubility should always be consulted before reconstituting small molecules.
  a. Reconstitute Thiazovivin (Tocris, 3845) at 20 mM using DMSO. Thiazovivin is a ROCK inhibitor.^41^
  b. Reconstitute CHIR99021 (Tocris, 4423) at 10 mM using DMSO. CHIR99021 is a GSK3 inhibitor,^42^ and therefore activates WNT signaling.
  c. Reconstitute DMH1 (Tocris, 4126) at 10 mM using DMSO. DMH1 is a BMP pathway inhibitor.^43^
  d. Reconstitute Forskolin (Tocris, 1099) at 10 mM using DMSO. Forskolin is an adenylate cyclase activator that elevates intracellular cAMP levels.^44^
  e. Reconstitute GDC0941 (Cellagen Technology, C4321) at 10 mM using DMSO. GDC0941 is a PI3K inhibitor.^45^
  f. Reconstitute SB505124 (Tocris, 3263) at 10 mM using DMSO. SB505124 is a TGF-β pathway inhibitor.^46^
  g. Reconstitute SR1 (Cellagen, C7710-1s) at 10 mM using DMSO. SR1 is an aryl hydrocarbon receptor (AHR) inhibitor that helps to maintain human hematopoietic stem and progenitor cells in an undifferentiated state.^47^
  h. Reconstitute TTNPB (Tocris, 0761) at 10 mM using DMSO. TTNPB is a retinoic acid receptor agonist.^48^
  i. Reconstitute UM171 (MedChemExpress, HY-12878) at 10 mM using DMSO. UM171 degrades the CoREST-LSD1 protein complex,^49^ and thereby helps to maintain human hematopoietic stem and progenitor cells in an undifferentiated state.^50^
    ***Note:*** Our laboratory has not tested UM171 from this supplier, but we list it as a potential substitute for our past supplier (Apex Bio), which no longer carries UM171.
  j. Reconstitute UNC0638 (Tocris, 4343) at 10 mM using DMSO. UNC0638 is a G9A/GLP H3K9 methyltransferase inhibitor^51^ that helps to maintain hematopoietic stem and progenitor cells in an undifferentiated state.^52^^,^^53^
  k. Reconstitute UNC1999 (Tocris, 4904) at 10 mM using DMSO. UNC1999 is a Polycomb Repressive Complex 2 (PRC2) methyltransferase inhibitor.^54^
  l. Reconstitute XAV939 (Tocris, 3748) at 10 mM using DMSO. XAV939 is a tankyrase inhibitor, and therefore inhibits WNT signaling.^55^
  m. Aliquot small molecules into 1.5 mL microcentrifuge tubes.
    ***Note:*** Aliquots should be prepared with reasonable working volumes, as repeated freeze/thaw cycles are detrimental to small molecule stability and should be avoided. When removing small molecules from −20°C for use, aliquots should be visually inspected to ensure that small molecules are still fully reconstituted (i.e., no solid particles visible).
  n. Dilute 10 mM TTNPB stocks one thousand-fold in DMSO to generate a 10 μM working stock.
    ***Note:*** TTNPB is used at very low concentrations in lateral mesoderm and artery induction media in this protocol, so 10 μM working stocks are more experimentally convenient. 10 μM TTNPB stocks can be stored −20°C but should be discarded after several freeze/thaw cycles, as dilute stocks may have limited long-term stability.
  o. Dilute 10 mM UM171 stocks ten-fold in DMSO to generate a 1 mM working stock.
    ***Note:*** UM171 is used at very low concentrations in hematopoietic progenitor induction medium in this protocol, so 1 mM working stocks are more experimentally convenient. 1 mM UM171 stocks can be stored at −20°C but should be discarded after several freeze/thaw cycles, as dilute stocks may have limited long-term stability.
4. Reconstitute 2-phospho-ascorbic acid (“AA2P”; Sigma, 49752) at 100 mg/mL in sterile water. AA2P is a stabilized form of vitamin C (ascorbic acid).
5. Reconstitute insulin (Sigma, 11376497001) at 10 mg/mL in sterile water.

**CRITICAL:** Insulin should not be reconstituted in PBS, as its solubility in PBS is poor.

**Table:** Reconstitution of growth factors and small molecules needed for differentiation

Reagent	Stock concentration	Diluent
**Activin A** (R&D Systems, 338-AC)	50 μg/mL	PBS + 0.1% BSA
**BMP4** (R&D Systems, 314-BP)	50 μg/mL	PBS + 0.1% BSA
**FGF2** (R&D Systems, 233-FB)	100 μg/mL	PBS + 0.1% BSA
**LIF** (R&D Systems, 7734-LF)	100 μg/mL	PBS + 0.1% BSA
**OSM** (R&D Systems, 295-OM)	100 μg/mL	PBS + 0.1% BSA
**VEGF-A** (R&D Systems, 293-VE)	100 μg/mL	PBS + 0.1% BSA
**Thiazovivin** (Tocris, 3845)	20 mM	DMSO
**CHIR99021** (Tocris, 4423)	10 mM	DMSO
**DMH1** (Tocris, 4126)	10 mM	DMSO
**Forskolin** (Tocris, 1099)	10 mM	DMSO
**GDC0941** (Cellagen Technology, C4321)	10 mM	DMSO
**SB505124** (Tocris, 3263)	10 mM	DMSO
**SR1** (Cellagen, C7710-1s)	10 mM	DMSO
**TTNPB** (Tocris, 0761)	10 mM (∗Dilute this concentrated stock to make a 10 μM TTNPB working stock)	DMSO
**UM171** (MedChemExpress, HY-12878)	10 mM (∗Dilute this concentrated stock to make a 1 mM UM171 working stock)	DMSO
**UNC0638** (Tocris, 4343)	10 mM	DMSO
**UNC1999** (Tocris, 4904)	10 mM	DMSO
**XAV939** (Tocris, 3748)	10 mM	DMSO
**2-phospho-ascorbic acid** (“AA2P”; Sigma, 49752)	100 mg/mL	H_2_O
**Insulin** (Sigma, 11376497001)	10 mg/mL	H_2_O

Storage: In our labs’ experience, reconstituted small molecules and recombinant growth factor proteins can typically be stored at −20°C for at least 1 year when stored at the listed concentrations; however, manufacturer’s recommendations for shelf life and storage should be consulted for each individual small molecule or growth factor. Additionally, aliquots should be visually inspected before use and repeated freeze/thaw cycles should be avoided.

**CRITICAL:** We recommend reconstituting growth factors and small molecules specifically at the concentrations listed above. Growth factors and small molecules reconstituted at higher concentrations have greater long-term stability.

### Coat plates with Geltrex basement membrane

**Timing: 40 min**

This section describes how to prepare cell culture plates with Geltrex basement membrane. hPSCs cannot adhere directly to cell culture plates, so basement membrane must be added to coat cell culture plates prior to cell seeding. We typically use Geltrex as a cost-efficient option^1^^,^^40^^,^^56^^,^^57^^,^^58^^,^^59^; however, Vitronectin^39^ may be substituted if a fully-defined basement membrane is desired. In our experience, Geltrex and Vitronectin are comparably effective in supporting cell differentiation and yield similar outcomes regarding the final differentiation purity.

***Note:*** Upon receipt, Geltrex should be aliquoted so that repeated freeze/thaw cycles may be avoided. Detailed methods to prepare Geltrex aliquots have been described elsewhere.^40^ New experimenters may wish to review these methods; alternatively, they could consult manufacturer’s recommendations for Geltrex storage and usage.

**CRITICAL:** Geltrex is temperature-sensitive. Always keep Geltrex cold and avoid prolonged exposure to ambient room temperature (20°C–22°C).

**Table:** 50 mL of 1% Geltrex

Reagent	Final concentration	Amount
**DMEM/F12** (Thermo Fisher, 11320033)	1X	50 mL
**Geltrex** (Thermo Fisher, A1413201)	1% v/v	500 μL

Storage: Diluted Geltrex can be stored at 4°C for 2 weeks (v/v = volume by volume).

6. Retrieve Geltrex (Thermo Fisher, A1413201) aliquots stored at −20°C and briefly thaw at 20°C–22°C. Then, add 500 μL of Geltrex to 50 mL of cold (4°C) DMEM/F12. Pipette mix gently to create a 1% Geltrex working stock.

***Note:*** For best results, 1% Geltrex should be left to dissolve at 4°C for 12–24 h before coating plates. Coating plates immediately after diluting Geltrex can sometimes lead to the appearance of small undissolved Geltrex clumps on the plate.

***Note:*** At this stage, an experimenter should determine what cell culture plate format best suits their needs. Our lab typically seeds hPSCs into 10 cm dishes for initial cell seeding, as later in differentiation, large numbers of cells are needed to resplit and re-seed cells at extremely high density.

7. Once 1% Geltrex has dissolved, work quickly to dispense 5 mL of Geltrex in the center of each 10 cm dish with a serological pipette.
8. Swirl each plate gently to spread the Geltrex over the entire surface of the dish. Visually inspect to ensure that even coating has been achieved. If there are any un-coated areas, pipette a small amount of Geltrex directly on to those areas and swirl gently once more.
9. Incubate Geltrex-coated dishes at 37°C for at least 30 min or up to 48 h.

### Prepare mTeSR plus human pluripotent stem cell medium

**Timing: around 12 h (to thaw mTeSR Plus supplement)**

This section describes how to prepare mTeSR Plus cell culture medium to grow hPSCs while maintaining cells in an undifferentiated state. While we typically use mTeSR Plus, other media (such as Essential 8 [E8] media^39^) may be used to culture hPSCs. In our experience, mTeSR Plus and E8 are comparably effective in supporting cell maintenance and yield similar outcomes regarding the final differentiation purity.

***Note:*** Each 500 mL unit of mTeSR Plus (STEMCELL Technologies, 100–0276) comprises two separate components: 400 mL of liquid basal medium (which should be stored at 4°C) and 100 mL of a frozen 5X supplement (which should be stored at −20°C).

10. Thaw the 100 mL bottle of 5X mTeSR Plus supplement at 4°C (typically takes 12–16 h).
11. Retrieve the 400 mL bottle of mTeSR Plus basal medium and add the thawed 100 mL of 5X mTeSR Plus supplement. Mix thoroughly.
12. Optionally, add 5 mL of 100X penicillin/streptomycin (Thermo Fisher Scientific, 15-140-122). This fully supplemented mTeSR medium (mTeSR Plus supplement + mTeSR Plus basal medium + penicillin/streptomycin) is hereafter referred to as “mTeSR Plus.”

***Note:*** Adding penicillin/streptomycin is optional but recommended, as penicillin/streptomycin is a prophylactic against bacterial contamination. We have not found that addition of penicillin/streptomycin impairs hPSC maintenance or differentiation.

**CRITICAL:** mTeSR Plus medium is thermally sensitive. Do not leave mTeSR Plus medium at temperatures greater than 4°C for prolonged amounts of time.

**Table:** 500 mL of mTeSR Plus + 1% penicillin/streptomycin (abbreviated “mTeSR Plus” for brevity)

Reagent	Final concentration	Amount
**mTeSR Plus basal medium** (STEMCELL Technologies, 100–0276)	1X	400 mL
**mTeSR Plus, 5X supplement** (STEMCELL Technologies, 100–0276)	1X	100 mL
**Penicillin/streptomycin** (Thermo Fisher Scientific, 15-140-122)	1% v/v	5 mL

Storage: mTeSR Plus + penicillin/streptomycin can be stored at 4°C for 1 month (v/v = volume by volume).

### Prepare media for initial seeding of hPSCs (for day 0 of differentiation)

**Timing: 10 min**

This section describes how to prepare media for initial seeding of hPSCs. When seeding hPSCs for differentiation, we first dissociate them into single cells.^40^ When hPSCs are dissociated to single cells they must be plated in media supplemented with a ROCK inhibitor (Thiazovivin)^41^ in order to survive. We recommend using Thiazovivin at 1 μM in the same media used to culture undifferentiated hPSCs prior to differentiation (e.g., mTeSR Plus, E8, etc.).

**Table:** 120 mL of mTeSR Plus + 1 μM Thiazovivin

Reagent	Final concentration	Amount
**mTeSR Plus** (STEMCELL Technologies, 100–0276)	1X	120 mL
**Thiazovivin, 20 mM stock** (Tocris, 3845)	1 μM	6 μL

Storage: mTeSR Plus + 1 μM Thiazovivin can be stored at 4°C for 1 month (v/v = volume by volume).

13. Thaw 20 mM Thiazovivin stock, and then add 6 μL Thiazovivin to 120 mL of mTeSR Plus medium (mTeSR Plus + 1% penicillin/streptomycin, as described above) and mix well to create an mTeSR Plus + 1 μM Thiazovivin solution.

***Note:*** We use Thiazovivin because it is more potent than the widely-used ROCK inhibitor Y-27632. Besides ROCK, Y-27632 also non-specifically inhibits other kinases, such as PRK2, RSK2, and AMPK.^60^

### Prepare CDM2 and CDM3 basal media for differentiation

**Timing: 12 h (to thaw KnockOut Serum Replacement)**

This section describes how to prepare the CDM2 and CDM3 basal media used for differentiation of hPSCs into hematopoietic progenitors. Chemically Defined Medium 2 (CDM2)^1^^,^^40^^,^^56^^,^^57^^,^^58^^,^^59^ is the basal medium for the first three steps of differentiation (generating posterior primitive streak, lateral mesoderm, and artery endothelial cells). CDM2 consists of 50% IMDM and 50% F12 (nutrient-rich basal media) supplemented with concentrated lipids (a cellular energy source), monothioglycerol (an antioxidant), insulin and transferrin (which support cell proliferation), polyvinyl alcohol (a synthetic polymer that stabilizes proteins in media), and penicillin/streptomycin (prophylactic antibiotics). Chemically Defined Medium 3 (CDM3)^1^^,^^56^ is the basal media for the last two steps of differentiation (generating hemogenic endothelial cells and hematopoietic progenitors). CDM3 consists of 45% IMDM and 45% F12 supplemented with concentrated lipids, polyvinyl alcohol, and penicillin/streptomycin, and 10% KnockOut Serum Replacement (Thermo Fisher, 10828010). Exogenous monothioglycerol, insulin, or transferrin is not added to CDM3, because KnockOut Serum Replacement already contains antioxidants, insulin, and transferrin.^61^ Both CDM2 and CDM3 are serum-free and should be supplemented with specific recombinant growth factor proteins and small molecule chemical compounds to create the differentiation media used for each differentiation step.

**Table:** 1000 mL of CDM2 basal medium

Reagent	Final concentration	Amount
**IMDM +GlutaMAX +HEPES +Sodium Bicarbonate** (Thermo Fisher, 31980-097)	50% v/v	490 mL
**F12 + GlutaMAX** (Thermo Fisher, 31765-092)	50% v/v	490 mL
**Polyvinyl alcohol** (Sigma, P8136)	1 mg/mL	1 g
**Concentrated lipids** (Thermo Fisher, 11905-031)	1% v/v	10 mL
**Monothioglycerol/1-thioglycerol, 11.5M stock** (Sigma, M6145)	450 μM	39.13 μL
**Insulin, 10 mg/mL stock** (Sigma, 11376497001)	0.7 μg/mL	70 μL
**Transferrin, 30 mg/mL stock** (Sigma, 10652202001)	15 μg/mL	500 μL
**Penicillin/streptomycin** (Thermo Fisher, 15140163)	1% v/v	10 mL

Storage: CDM2 basal medium can be stored at 4°C for 2 months (v/v = volume by volume).

14. Tare a 500 mL sterile beaker on a weighing scale. Add 1 g of polyvinyl alcohol powder to the beaker.

***Note:*** This step can be performed under non-sterile conditions outside of the cell culture hood, as CDM2 will be sterilely-filtered later on.

15. Add 200 mL of IMDM and a sterile magnetic stir bar to the beaker. Place the beaker on a hot plate with magnetic stirring capacity, and apply gentle warming and magnetic stirring.

***Note:*** Polyvinyl alcohol has poor solubility and thus requires a considerable amount of time to dissolve fully. Additionally, it cannot dissolve readily without heat and stirring. The amount of time required to dissolve polyvinyl alcohol may vary depending on the level of heat used to warm the IMDM solvent. We do not recommend using high volumes of IMDM, which take longer to heat. We recommend using moderate heat and monitoring the IMDM/polyvinyl alcohol solution to ensure it does not overheat and boil. Additionally, we do not recommend attempting to dissolve multiple grams of polyvinyl alcohol at once, as larger amounts of polyvinyl alcohol may not dissolve well in the recommended 200 mL of IMDM. The IMDM/polyvinyl alcohol solution should be visually inspected to ensure full dissolution prior to use in CDM2.

16. In a sterile cell culture hood, add all CDM2 media components to a sterile 0.22 μm filter unit and vacuum filter to sterilize.
  a. Allow the IMDM/polyvinyl alcohol solution to cool slightly, then add it to the filter unit.
  b. Thoroughly mix the concentrated lipids with a 10 mL serological pipette, then add 10 mL to the filter unit.
  c. Add all other CDM2 media components to the filter unit. Mix thoroughly before use.

**Table:** 1000 mL of CDM3 basal medium

Reagent	Final concentration	Amount
**IMDM +GlutaMAX +HEPES +Sodium Bicarbonate** (Thermo Fisher, 31980-097)	45% v/v	440 mL
**F12 + GlutaMAX** (Thermo Fisher, 31765-092)	45% v/v	440 mL
**KnockOut Serum Replacement** (Thermo Fisher, 10828010)	10% v/v	100 mL
**Polyvinyl alcohol** (Sigma, P8136)	1 mg/mL	1 g
**Concentrated lipids** (Thermo Fisher, 11905-031)	1% v/v	10 mL
**Penicillin/streptomycin** (Thermo Fisher, 15140163)	1% v/v	10 mL

Storage: CDM3 basal medium can be stored at 4°C for 2 months (v/v = volume by volume).

17. Retrieve KnockOut Serum Replacement (Thermo Fisher, 10828010) from −20°C storage and thaw at 4°C (typically takes 12–16 h).
18. Follow steps 14–15 above to dissolve 1 g of polyvinyl alcohol in 200 mL of IMDM.
19. In a sterile cell culture hood, add all CDM3 media components to a sterile 0.22 μm filter unit and vacuum filter to sterilize.
  a. Allow the IMDM/polyvinyl alcohol solution to cool slightly, then add it to the filter unit.
  b. Thoroughly mix the concentrated lipids with a 10 mL serological pipette, then add 10 mL to the filter unit.
  c. Add all other CDM3 media components to the filter unit. Mix thoroughly before use.

### Prepare posterior primitive streak differentiation medium (for days 1 and 2 of hPSC differentiation)

**Timing: 10 min**

This section describes how to prepare posterior primitive streak differentiation medium, which is used for the first step of differentiation. In the developing embryo, the primitive streak forms the endoderm and mesoderm germ layers.^62^ However, there are different regions of the primitive streak, including the anterior, middle, and posterior primitive streak.^62^ The posterior primitive streak exclusively expresses *Hoxa5-Hoxa10*,^63^^,^^64^ which are also expressed by future HSCs.^12^^,^^34^^,^^36^ To induce posterior primitive streak cells *in vitro*, we simultaneously provide agonists of the BMP, FGF, and WNT signaling pathways.^1^ FGF and WNT broadly induce primitive streak identity, whereas BMP specifically induces posterior primitive streak.^58^^,^^59^^,^^65^^,^^66^^,^^67^ At this stage, we do not provide an exogenous TGF-β agonist, which instead instructs anterior primitive streak formation.^58^^,^^59^ After 2 days of differentiation, the resultant hPSC-derived posterior primitive streak cells express pan-primitive streak transcription factors (*BRACHYURY* and *MIXL1*) alongside posterior primitive streak transcription factors (*CDX2*, *CDX4*, *HOXA5*, *HOXA7*, *HOXA9*, and *HOXA10*).^1^

**Table:** 100 mL of posterior primitive streak differentiation medium (for days 1 and 2 of hPSC differentiation)

Reagent	Mechanism	Stock concentration	Final concentration	Amount
**BMP4** (R&D Systems, 314-BP)	BMP activator	50 μg/mL	40 ng/mL	80 μL
**CHIR99021** (Tocris, 4423)	WNT activator	10 mM	6 μM	60 μL
**FGF2** (R&D Systems, 233-FB)	FGF activator	100 μg/mL	20 ng/mL	20 μL
**CDM2 basal medium** (generated as described above)	Basal medium	1X	1X	100 mL

Storage: Posterior primitive streak differentiation medium can be stored at 4°C for up to 3 days.

20. Thaw frozen aliquots of BMP4, CHIR99021, and FGF2, then add the indicated amounts of each factor into a 100 mL aliquot of CDM2.

***Note:*** For this step (and all following steps), we recommend using differentiation media within 3 days of preparation. We have not rigorously tested how long differentiation media remains stable at 4°C, nor whether it can be frozen and thawed.

**CRITICAL:** For this step (and all following steps), ensure that the stock concentration of each differentiation factor matches the concentration listed in the table above. Visually inspect all small molecules while they are still frozen to ensure that they are fully reconstituted and free of precipitates. Discard any small molecules containing visible precipitates, as they may no longer be usable.

**CRITICAL:** For this step (and all following steps), do not substitute the listed differentiation factors with other factors, even if they are reported to affect the same pathways.

### Prepare lateral mesoderm differentiation medium (for day 3 of hPSC differentiation)

**Timing: 20 min**

This section describes how to prepare lateral mesoderm differentiation medium, which is used for the second step of differentiation. *In vivo*, the lateral mesoderm gives rise to multiple cell-types, including endothelial cells (ECs).^68^^,^^69^^,^^70^ To achieve differentiation of hPSC-derived posterior primitive streak cells into lateral mesoderm cells within 24 h, we simultaneously provide a BMP activator, VEGF activator, cAMP/PKA pathway activator, RA activator, WNT inhibitor, TGF-β inhibitor, PI3K inhibitor, and a stabilized vitamin C analog (2-phospho-ascorbic acid; “AA2P”) in CDM2 basal medium.^1^ The developmental rationale for why this combination of signals was selected to generate lateral mesoderm cells is described in further detail elsewhere.^40^^,^^69^ However, here we emphasize four signaling pathways particularly important for lateral mesoderm specification. BMP activation and VEGF activation are crucial to differentiate primitive streak cells into lateral mesoderm.^57^^,^^59^^,^^71^^,^^72^^,^^73^ Concurrently, we block the WNT pathway to suppress paraxial mesoderm formation,^59^ and we also inhibit TGF-β signaling to block endoderm differentiation.^58^ Taken together, these signals direct differentiation of posterior primitive streak into lateral mesoderm, while blocking differentiation into extraneous *MSGN1+ TBX6+* paraxial mesoderm and *FOXA2+* endoderm.

**Table:** 50 mL of lateral mesoderm differentiation medium (for day 3 of hPSC differentiation)

Reagent	Mechanism	Stock concentration	Final concentration	Amount
**AA2P** (Sigma, 49752)	Stabilized vitamin C	100 mg/mL	200 μg/mL	100 μL
**VEGF** (R&D Systems, 293-VE)	VEGF activator	100 μg/mL	100 ng/mL	50 μL
**Forskolin** (Tocris, 1099)	Elevates cAMP	10 mM	10 μM	50 μL
**BMP4** (R&D Systems, 314-BP)	BMP activator	50 μg/mL	40 ng/mL	40 μL
**GDC0941** (Cellagen Technology, C4321)	PI3K inhibitor	10 mM	2.5 μM	12.5 μL
**SB505124** (Tocris, 3263)	TGF-β inhibitor	10 mM	2 μM	10 μL
**XAV939** (Tocris, 3748)	WNT inhibitor	10 mM	1 μM	5 μL
**TTNPB** (Tocris, 0761)	Retinoic acid analog	10 μM	0.5 nM	2.5 μL
**CDM2 basal medium** (generated as described above)	Basal medium	1X	1X	50 mL

Storage: Lateral mesoderm differentiation medium can be stored at 4°C for up to 3 days.

21. Thaw frozen aliquots of AA2P, VEGF, Forskolin, BMP4, GDC0941, SB505124, XAV939, and TTNPB, then add the indicated amounts of each factor into a 50 mL aliquot of CDM2.

**CRITICAL:** Before adding each differentiation factor, ensure that its stock concentration matches the concentration listed in the table above. Take special care to ensure that the 10 μM working stock of TTNPB is used (instead of the 10 mM concentrated stock).

**CRITICAL:** Do not substitute the listed differentiation factors with other factors, even if they are reported to affect the same pathways. For instance, replacing the TGF-β pathway inhibitor SB505124 with the alternative TGF-β pathway inhibitor A8301 will compromise differentiation, as A8301 also inhibits the VEGF receptor,^74^ and VEGF signaling is required for endothelial differentiation.

### Prepare artery endothelium differentiation medium (for day 4 of hPSC differentiation)

**Timing: 20 min**

This section describes how to prepare artery endothelium differentiation medium, which is used for the third step of differentiation. To generate artery endothelial cells (ECs), we simultaneously provide a VEGF agonist, TGF-β agonist, WNT inhibitor, BMP inhibitor, PI3K inhibitor, and a stabilized vitamin C analog (2-phospho-ascorbic acid; “AA2P”) in CDM2 basal medium.^1^ The developmental rationale for why this combination of signals was selected to generate artery ECs is described in further detail elsewhere.^40^^,^^69^ However, here we highlight the important roles of VEGF agonist^72^^,^^75^ and TGF-β agonist,^57^^,^^76^ both of which are vital for artery EC specification. At this differentiation step, we also block the WNT, BMP, and PI3K pathways to suppress the formation of unwanted cell-types, including *NKX2.5+* heart progenitors and *APLNR+ NR2F2+* vein ECs.^57^^,^^77^

**Table:** 50 mL of artery endothelium differentiation medium (for day 4 of hPSC differentiation)

Reagent	Mechanism	Stock concentration	Final concentration	Amount
**AA2P** (Sigma, 49752)	Stabilized vitamin C	100 mg/mL	200 μg/mL	100 μL
**VEGF** (R&D Systems, 293-VE)	VEGF activator	100 μg/mL	100 ng/mL	50 μL
**Activin A** (R&D Systems, 338-AC)	TGF-β inhibitor	50 μg/mL	15 ng/mL	15 μL
**GDC0941** (Cellagen Technology, C4321)	PI3K inhibitor	10 mM	2.5 μM	12.5 μL
**XAV939** (Tocris, 3748)	WNT inhibitor	10 mM	1 μM	5 μL
**TTNPB** (Tocris, 0761)	Retinoic acid analog	10 μM	0.5 nM	2.5 μL
**DMH1** (Tocris, 4126)	BMP inhibitor	10 mM	250 nM	1.25 μL
**CDM2 basal medium** (generated as described above)	Basal medium	1X	1X	50 mL

Storage: Artery endothelium differentiation medium can be stored at 4°C for up to 3 days.

22. Thaw frozen aliquots of AA2P, VEGF, Activin A, GDC0941, XAV939, TTNPB, and DMH1, then add the indicated amounts of each factor into a 50 mL aliquot of CDM2.

**CRITICAL:** Before adding each differentiation factor, ensure that its stock concentration matches the concentration listed in the table above. Take special care to ensure that the 10 μM working stock of TTNPB is used (instead of the 10 mM concentrated stock).

**CRITICAL:** Do not substitute the listed differentiation factors with other factors, even if they are reported to affect the same pathways. For instance, replacing the BMP pathway inhibitor DMH1 with the alternative BMP pathway inhibitor LDN193189 will compromise differentiation, as LDN193189 inhibits both the VEGF and TGF-β receptors,^74^ and VEGF signaling is required for EC differentiation.

### Prepare hemogenic endothelium differentiation medium (for days 5 through 7 of hPSC differentiation)

**Timing: 20 min**

This section describes how to prepare hemogenic endothelium differentiation medium, which is used for the fourth step of differentiation. To generate hemogenic ECs, we simultaneously (1) activate the GP130 pathway, (2) activate the cAMP/PKA pathway, (3) inhibit the TGF-β pathway, (4) inhibit Polycomb Repressive Complex 2 (PRC2), and (5) activate the NOTCH pathway. The developmental rationale for each of these signaling manipulations is provided here. First, the GP130 pathway ligand OSM is important for the development and expansion of hematopoietic stem and progenitor cells *in vivo*.^78^^,^^79^^,^^80^ Second, prostaglandin E2 and shear stress are important for hematopoietic stem and progenitor cell formation *in vivo*, and both activate cAMP/PKA signaling.^81^^,^^82^ Third, TGF-β signaling often suppresses hematopoietic development.^25^^,^^83^^,^^84^ Fourth, the PRC2 methyltransferase *Ezh1* suppresses HSC emergence *in vivo*, such that HSCs precociously emerge in *Ezh1*^−/−^ mouse embryos.^15^ Finally, the NOTCH pathway is crucial for HSC formation *in vivo*, as underscored by the complete absence of functional HSCs in *Notch1*^−/−^ mouse embryos.^85^^,^^86^

**Table:** 50 mL of hemogenic endothelium differentiation medium (for days 5 through 7 of hPSC differentiation)

Reagent	Mechanism	Stock concentration	Final concentration	Amount
**Forskolin** (Tocris, 1099)	Elevates cAMP	10 mM	10 μM	50 μL
**SB505124** (Tocris, 3263)	TGF-β inhibitor	10 mM	2 μM	10 μL
**LIF** (R&D Systems, 7734-LF)	GP130 pathway activator	100 μg/mL	20 ng/mL	10 μL
**OSM** (R&D Systems, 295-OM)	GP130 pathway activator	100 μg/mL	10 ng/mL	5 μL
**UNC1999** (Tocris, 4904)	PRC2 inhibitor	10 mM	1 μM	5 μL
**CDM3 basal medium** (generated as described above)	Basal medium	1X	1X	50 mL

Storage: Hemogenic endothelium differentiation medium can be stored at 4°C for up to 3 days.

23. Thaw frozen aliquots of Forskolin, SB505124, LIF, OSM, and UNC1999, then add the indicated amounts of each factor into a 50 mL aliquot of CDM3.

### Prepare Vitronectin + DLL4-E12 basement membrane (for days 5 through 10 of hPSC differentiation)

**Timing: 10 min**

This section describes how to prepare NOTCH-coated plates, which are used for the fourth and fifth steps of differentiation. The NOTCH pathway cannot be readily activated by soluble ligands present in the culture medium.^86^ In fact, soluble NOTCH ligands often inhibit NOTCH signaling, owing to the requirement for mechanical force to activate NOTCH receptor signaling.^86^ Consequently, we do not add a soluble NOTCH ligand in hemogenic EC medium, but rather we immobilize NOTCH ligands to the surface of the cell-culture plate. To this end, we mix Vitronectin basement membrane (Thermo Fisher, A14700) with the NOTCH ligand DLL4-E12.^87^ DLL4-E12 is a mutant version of DLL4 that binds the NOTCH1 receptor with over 200 times increased affinity relative to wild-type DLL4.^87^ While we exclusively used DLL4-E12 in our original study,^1^ we have found that DLL4-E12 can be substituted with commercially-available, wild-type DLL4 (R&D Systems, 10185-D4) (Figure 2).

**Table:** 50 mL of Vitronectin + DLL4-E12

Reagent	Final concentration	Amount
**PBS**	1X	50 mL
**Vitronectin [VTN-N], 0.5 mg/mL stock** (Thermo Fisher, A14700)	10 μg/mL	1 mL
**DLL4-E12**	20 nM	Volume depends on DLL4-E12 concentration, which varies by batch (for example, add 20 μL of 50 μM DLL4-E12 to 50 mL of VTN-N in PBS to achieve a final concentration of 20 nM)

Storage: Vitronectin + DLL4-E12 can be stored at 4°C for up to 1 month.

24. Retrieve Vitronectin (Thermo Fisher, A14700) from storage at −80°C and thaw at 20°C–22°C. Then, add 1 mL of Vitronectin to 50 mL of PBS to create a 10 μg/mL Vitronectin working stock.
25. Retrieve DLL4-E12 from storage at −20°C and thaw at 20°C–22°C. Then, add DLL4-E12 to the Vitronectin working stock to a final concentration of 20 nM.

### Prepare hematopoietic progenitor differentiation media (for days 8 through 10 of hPSC differentiation)

**Timing: 20 min**

This section describes how to prepare hematopoietic progenitor differentiation medium, which is used for the fifth and final step of differentiation. To generate hematopoietic progenitors, we simultaneously (1) activate the cAMP/PKA pathway, (2) inhibit the TGF-β pathway, (3) inhibit Polycomb Repressive Complex 2 (PRC2), (4) inhibit G9A/GLP, (5) inhibit aryl hydrocarbon receptor (AHR), (6) inhibit the CoREST-LSD1 complex, and (7) activate the NOTCH pathway. The rationale for activating cAMP/PKA, inhibiting TGF-β, inhibiting PRC2, and activating NOTCH was described in the preceding section. Additionally, in the final step of differentiation we added inhibitors of AHR,^47^ CoREST-LSD1,^49^^,^^50^ and G9A/GLP,^52^^,^^53^ because each of these inhibitors helps to maintain undifferentiated hematopoietic stem and progenitor cells *ex vivo*.

**Table:** 50 mL of hematopoietic progenitor differentiation media (for days 8 through 10 of hPSC differentiation)

Reagent	Mechanism	Stock concentration	Final concentration	Amount
**Forskolin** (Tocris, 1099)	Elevates cAMP	10 mM	10 μM	50 μL
**SB505124** (Tocris, 3263)	TGF-β inhibitor	10 mM	2 μM	10 μL
**UNC1999** (Tocris, 4904)	PRC2 inhibitor	10 mM	1 μM	5 μL
**SR1** (Cellagen, C7710-1s)	GP130 pathway activator	10 mM	750 nM	3.75 μL
**UM171** (MedChemExpress, HY-12878)	GP130 pathway activator	1 mM	75 nM	3.75 μL
**UNC0638** (Tocris, 4343)	G9A/GLP pathway inhibitor	10mM	500nM	2.5 μL
**CDM3 basal medium** (generated as described above)	Basal medium	1X	1X	50 mL

Storage: Hematopoietic progenitor differentiation medium can be stored at 4°C for up to 3 days.

26. Thaw frozen aliquots of Forskolin, SB505124, UNC1999, SR1, UM171, and UNC0638, then add the indicated amounts of each factor into a 50 mL aliquot of CDM3.

**CRITICAL:** Before adding each differentiation factor, ensure that its stock concentration matches the concentration listed in the table above. Take special care to ensure that the 1 mM working stock of UM171 is used (instead of the 10 mM concentrated stock).

### Culture undifferentiated hPSCs

**Timing: variable**

Detailed methods to thaw, maintain, passage, and freeze undifferentiated hPSCs have been described elsewhere.^40^ We recommend that new experimenters familiarize themselves with these methods before proceeding. We recommend growing hPSCs in 6-well plates or 10-cm dishes to generate sufficient numbers of hPSCs for downstream differentiation. Typically, a confluent 6-well plate of H1 or H7 hPSCs yields ∼2–3 million cells, while a confluent 10-cm dish of H1 or H7 hPSCs yields ∼20–30 million cells.

27. Maintain undifferentiated hPSCs on Geltrex-coated plates in mTeSR Plus, passaging when necessary. When a sufficient number of cells is obtained, proceed with differentiation.

## Key resources table

**Table:** 

REAGENT or RESOURCE	SOURCE	IDENTIFIER
**Antibodies**		

Anti-CD34 eFluor 450 antibody (clone 4H11), used at 1:100	BD Biosciences	48-0349-41
Anti-CD38 Brilliant Violet 510 antibody (clone HIT2), used at 1:100	BD Biosciences	563251
Anti-CD45 Alexa Fluor 488 antibody (clone H130), used at 1:100	BioLegend	304017
Anti-CD45RA Brilliant Violet 605 antibody (clone HI100), used at 1:100	BioLegend	304134
Anti-CD90 Brilliant Violet 421 antibody (clone 5E10), used at 1:100	BD Biosciences	562556

**Chemicals, peptides, and recombinant proteins**		

mTeSR Plus medium	STEMCELL Technologies	100-0276
Essential 8 medium	Thermo Fisher Scientific	A1517001
Penicillin/streptomycin	Thermo Fisher Scientific	15-140-122
Geltrex LDEV-free, hESC-qualified, reduced growth factor basement membrane matrix	Thermo Fisher Scientific	A1413302
Vitronectin	Thermo Fisher Scientific	A14700
DLL4-E12	Vincent Luca’s laboratory, Moffitt Cancer Center	Luca et al. ^87^
DLL4	R&D Systems	10185-D4
Accutase - Enzyme Cell Detachment medium	Thermo Fisher Scientific	00-4555-56
TrypLE Express Enzyme (1X)	Thermo Fisher Scientific	12604013
CryoStor CS10	STEMCELL Technologies	07930
DMEM/F12 + GlutaMAX	Thermo Fisher Scientific	10565042
IMDM + GlutaMAX	Thermo Fisher Scientific	31980-097
F12 + GlutaMAX	Thermo Fisher Scientific	31765-092
Polyvinyl alcohol	Sigma	P8136-250G
Chemically defined lipid concentrate	Thermo Fisher Scientific	11905-031
1-thioglycerol (Monothioglycerol)	Sigma	M6145-100ML
Human transferrin	Sigma	10652202001
KnockOut Serum Replacement	Thermo Fisher Scientific	10828010
Recombinant human insulin	Sigma	11376497001
Recombinant Activin A	R&D Systems	338-AC-500/CF
Recombinant human BMP4	R&D Systems	314-BP-050
Recombinant human FGF2	R&D Systems	233-FB-01M
Recombinant human LIF	R&D Systems	7734-LF
Recombinant human OSM	R&D Systems	295-OM
Recombinant human VEGF-A	R&D Systems	293-VE-0500
Thiazovivin	Tocris	3845
CHIR99201	Tocris	4423
DMH1	Tocris	4126
Forskolin	Tocris	1099
GDC-0941	Cellagen Technology	C4321-25
SB505124	Tocris	3263
SR1	Cellagen Technology	C7710-1s
TTNPB	Tocris	0761
UM171	MedChemExpress	HY-12878
UNC0638	Tocris	4343
UNC1999	Tocris	4904
XAV939	Tocris	3748
Ascorbic acid-2-phosphate (“AA2P″)	Sigma	49752-10G
DMSO (dimethyl sulfoxide)	Sigma	D2650
Trypan blue	Thermo Fisher Scientific	T10282
Propidium iodide, used at 1:1,000	Cytek Bio	13-6990-T200

**Critical commercial assays**		

Steriflip 50 mL volume filter, 0.22 μm pore size	Thermo Fisher Scientific	SE1M179M6
Stericup Quick Release-GV sterile vacuum filtration system, 1000 mL volume, 0.22 μm pore size	Sigma	S2GVU10RE
Bovine albumin fraction V (7.5% solution)	Thermo Fisher Scientific	15260037
UltraPure DNase/RNase-free distilled water	Thermo Fisher Scientific	10977023
PBS, pH 7.4	Thermo Fisher Scientific	10010049

**Experimental models: Cell lines**		

Human H1 hESCs, XY genotype, Central European ethnicity	WiCell	WA01
Human H7 hESCs, XX genotype, Middle Eastern ethnicity	WiCell	WA07
Human 1157 hiPSCs, XX genotype, Caucasian ethnicity	Boston Children’s Hospital	Sugimura et al. ^16^
Human WTC11 hiPSCs, XY genotype, Japanese ethnicity	Coriell Institute for Medical Research	GM25256, Kreitzer et al. ^38^

**Oligonucleotides**		

YWHAZ Forward primer: GAGCTGGTTCAGAAGGCCAAAC Reverse primer: CCTTGCTCAGTTACAGACTTCATGC A	Fowler and Zheng et al.^1^	N/A
CD43 Forward primer: CACTTCAATAACAAGTGACCCTAA GG Reverse primer: TGGTAGGTTGTTGGCTCAGGTA	Fowler and Zheng et al.^1^	N/A
HLF Forward primer: CTGGGGCCTACCTTATGGGA Reverse primer: GGGGAATGCCATTTTCTGACA	Fowler and Zheng et al.^1^	N/A
HOXA5 Forward primer: AAACTGTGACTCCAAGCGGT Reverse primer: GAGCCACTTCCAGAGTTCGT	Fowler and Zheng et al.^1^	N/A
HOXA7 Forward primer: AGGAGTTCCACTTCAACCGC Reverse primer: CAGTCGGACCTTCGTCCTTAT	Fowler and Zheng et al.^1^	N/A
HOXA9 Forward primer: TTGCACCAGACGAACAGTGA Reverse primer: GCCCAATGGCGGTTTCATAG	Fowler and Zheng et al.^1^	N/A
HOXA10 Forward primer: CTGGTTTCAGAACCGCAGGA Reverse primer: AGATGTAACGGCCCAGGAGA	Fowler and Zheng et al.^1^	N/A

## Step-by-step method details

### Dissociate hPSCs into single cells using Accutase, and seed them for differentiation

**Timing: 1 h**

This section describes how to seed hPSCs for differentiation. First, hPSCs are dissociated into single cells using Accutase and plated onto Geltrex-coated plates. As discussed above, Accutase-dissociated hPSCs must be plated in media supplemented with a ROCK inhibitor (Thiazovivin)^41^ in order to survive. The recipe to prepare mTeSR Plus + 1 μM Thiazovivin has been described above. If seeding hPSCs into 10 cm dishes, 10 mL of mTeSR Plus + 1 μM Thiazovivin should be prepared for each dish, with some excess available if needed.

**CRITICAL:** Optimizing initial cell seeding densities is critical for achieving efficient differentiation. Initial seeding density should be re-optimized for each new hPSC line, cell culture plate format, and experimenter. Therefore we strongly recommend that a new experimenter should test a range of cell seeding densities. For mTeSR Plus-grown hPSCs, we recommend seeding 40,000–50,000 hPSCs/cm^2^ (i.e., ∼2,250,000–2,750,000 hPSCs per 10 cm dish). An experimenter should also try to seed cells above and below this recommended range.

A step-by-step protocol for dissociating hPSCs with Accutase is provided here.

1. Aspirate mTeSR Plus from largely-confluent hPSCs and add Accutase (Thermo Fisher, 00-4555-56) to dissociate cells. Incubate plates at 37°C for ∼5 min to dissociate cells.

***Note:*** Typically, add half the volume of Accutase that you would add of cell culture media suited for that type of cell culture well or dish. For instance, add at least 1 mL of Accutase per well of a 6-well plate or 5 mL per 10-cm dish to fully cover the surface.

***Note:*** Observe the well/plate throughout the Accutase incubation period. Within several minutes, most cells should detach from the bottom of the well/plate. If many colonies remain attached, extend the incubation period by a few minutes until most colonies are detached.

2. When near-complete cell detachment has occurred, transfer dissociated cells into a 50 mL conical tube. Add a small volume of DMEM/F12 to wash any remaining cells off of the well/plate.

***Note:*** This wash step may be repeated if cells remain attached to the plate after each wash.

3. Add additional DMEM/F12 to the conical tube. The volume of DMEM/F12 added should be sufficient to dilute the original volume of Accutase by at least 1:5. Next, centrifuge the tube at 500 × g for 5 min at 4°C to pellet hPSCs.
4. While the dissociated hPSCs are pelleting, prepare Geltrex-coated 10 cm dishes to receive hPSCs.
  a. Ensure that, as described above, dishes have been coated with Geltrex for a minimum of 30 min.
  b. Once Geltrex coating is complete, aspirate the Geltrex, taking care not to disturb the thin translucent film at the bottom of the dish.
  c. Then, add half a volume of mTeSR Plus + 1 μM Thiazovivin: for a 10 cm dish, add 5 mL of mTeSR Plus + 1 μM Thiazovivin.

***Note:*** Media should be added to the side wall of the dish to avoiding disrupting the Geltrex coating on the bottom of the dish.

**CRITICAL:** Thiazovivin is critical for the survival of hPSCs that have been dissociated into single cells.

5. After centrifugation is complete, carefully retrieve the 50 mL conical tube from the centrifuge and attempt to visualize the pellet. Carefully aspirate or decant the supernatant without disturbing the cell pellet.

***Note:*** Pellets may not be visible for smaller cell numbers.

6. Resuspend the cell pellet with mTeSR Plus + 1 μM Thiazovivin, preferably using a 5 mL or a 10 mL pipette tip. Triturate gently to break up any cell clumps, taking care not to over-triturate.

**CRITICAL:** It is critical to evenly resuspend the cell pellet to ensure an accurate cell count (see below step). However, it is equally critical to avoid over-triturating hPSCs when resuspending them, as over-trituration can lead to cell death.

7. After resuspending hPSCs in mTeSR Plus + 1 μM Thiazovivin, take an aliquot of cells for counting on the hemocytometer or automated cell counter.

***Note:*** Typically, a confluent 6-well plate of H1 or H7 hPSCs yields ∼2–3 million cells, while a confluent 10 cm dish of H1 or H7 hPSCs yields ∼20–30 million cells. Cells should be mixed with a viability stain such as Trypan Blue (Thermo Fisher, T10282) for counting. Only live cells (e.g., not stained by Trypan Blue) cells should be counted.

8. After cell counting is complete, adjust the volume of mTeSR Plus + 1 μM Thiazovivin to seed hPSCs at the desired density.
  a. For instance, if an experimenter wishes to seed 2.5 million hPSCs per 10 cm dish, they could resuspend hPSCs at a density of 500,000 cells/mL. Subsequently, they could seed 5 mL of cell suspension per 10 cm dish; each 10 cm dish should already contain 5 mL of mTeSR Plus + 1 μM Thiazovivin (as described above). In this scenario, each 10 cm dish would contain 2.5 million cells in 10 mL of media.

***Note:*** It is imperative to ensure that hPSCs are evenly resuspended, to ensure that equal numbers of hPSCs are seeded per recipient well.

9. Gently pipette mix to evenly disperse cells. Then, pipette 5 mL of the hPSC suspension from the 50 mL conical tube into recipient dishes. The recipient dishes should have been pre-coated with 5 mL of mTeSR Plus + 1 μM Thiazovivin.
  a. When adding the cell suspension to recipient dishes, do so briskly; if pipetted too slowly, cells may clump at the bottom of a serological pipette tip, leading to uneven seeding.
  b. Ensure that recipient dishes already contain 5 mL of mTeSR Plus + 1 μM Thiazovivin (as described above). It is important to ensure that dishes already contain some media. Otherwise, adding cell suspension directly to an empty dish can lead to uneven cell plating.
  c. We have observed that resuspending and dispersing cells in a greater volume (i.e., more than 2 mL) is beneficial for even cell seeding. If cells are resuspended in small volumes (i.e., less than 2 mL), seeding is sometimes uneven.
10. Immediately after seeding cells, evenly shake the plate in a cross pattern (left, then right; up, then down) several times to make sure clumps are evenly distributed across the dish. Quickly check under a microscope to ensure that cells are evenly distributed throughout the dish. If not, continue shaking the plate.

**CRITICAL:** To ensure even cell seeding, shake the plate as soon as possible after adding the cell suspension. hPSCs adhere to Geltrex rapidly, and once unevenly plated, it is impossible to dislodge cells and re-distribute them. Do not swirl the plate in a circular motion, as this will promote cell clumping at the center of the plate.

11. Return the cells to the 37°C incubator to recover overnight (16–24 h).

***Note:*** If stacking multiple dishes of hPSCs in the incubator, ensure that each dish is flat: if unevenly stacked, hPSCs will become asymmetrically distributed across the plate and form colonies of uneven size.

12. On the following day, use a microscope to observe the seeded cells.

**Figure 1:**
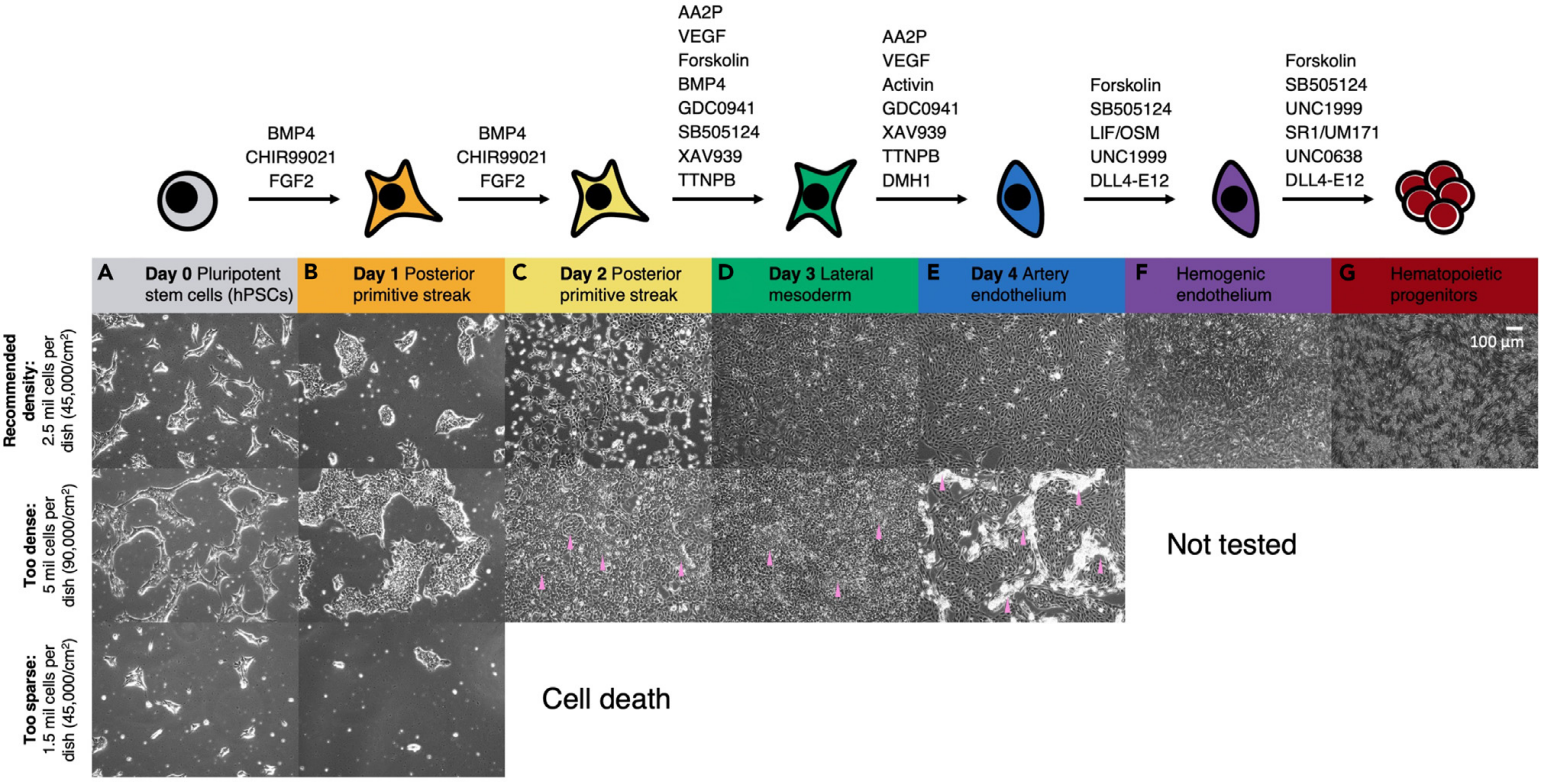
Morphology of H1 hPSCs being sequentially differentiated into hematopoietic progenitors at three different densities In this experiment, 2.5 million cells per 10 cm dish represents an ideal initial seeding density (top row). 5 million cells per 10 cm dish is too dense, and contaminating cell-types become visible between days 2–4 (middle row). 1.5 million cells per 10 cm dish is too sparse, and cells die by day 2 (bottom row). At each stage of differentiation, cultured cells undergo morphological changes, aiding visual tracking of differentiation. Scale = 100 μm. (A) Undifferentiated hPSCs were dissociated into single cells with Accutase, seeded on Geltrex-coated 10 cm dishes in mTeSR Plus + 1 μM Thiazovivin, and allowed to recover for 24 h. At this stage, hPSC colonies are tightly-packed and appear as spiky webs. (B) Day 1 posterior primitive streak cells are larger, more rounded, and more spread out. (C) Day 2 posterior primitive streak cells are triangular or star-like in shape. Cells should spread out and achieve semi-confluency at this stage (top row). They should not be fully confluent (middle row), as over-confluency can lead to the emergence of contaminating cell-types (arrows). (D) Day 3 lateral mesoderm cells remain star-shaped and form a near-confluent monolayer. If cells are over-confluent, large swaths of contaminating mesenchyme-like cells may be visible (arrows). (E) Day 4 arterial endothelium appears more rounded and form a near-confluent monolayer. If cells are over-confluent, large swaths of contaminating mesenchyme-like cells may be visible (arrows). (F) Day 5–7 hemogenic endothelium appears as a densely packed monolayer (Day 7 hemogenic endothelium shown). (G) Between Days 8–10, semiadherent cells begin to emerge (Day 10 hematopoietic progenitors shown).

***Note:*** There may be some degree of cell death, but in the presence of Thiazovivin substantial numbers of surviving cells should remain. If passaged properly, within 24 h post-seeding, hPSCs should have reformed into spiky “webs” of cells (Figure 1A). These hPSCs are now ready for differentiation, as described below.

### Differentiate hPSCs into hematopoietic progenitors

**Timing: 10 days**

This section describes how to differentiated hPSCs into *HLF+ HOXA+* hematopoietic progenitors (Figure 1). hPSCs are differentiated through the following intermediates: day 1–2 posterior primitive streak, day 3 lateral mesoderm, day 4 artery endothelium, day 5–7 hemogenic endothelium, and finally, day 8–10 hematopoietic progenitors. A critical event partway through differentiation occurs at the end of day 4 of differentiation: artery ECs are dissociated and re-seeded at extremely high density onto plates that are coated with both vitronectin and DLL4-E12. This serves two functions. First, we found that dissociating and re-seeding artery ECs at extremely high density is critical to subsequently produce hematopoietic progenitors.^1^ Second, we plate artery ECs onto plates that are immobilized with DLL4-E12, which is a powerful NOTCH pathway agonist that binds the NOTCH1 receptor with over 200 times increased affinity relative to wild-type DLL4.^87^ As aforementioned, NOTCH ligands must typically be immobilized to a solid surface to activate NOTCH signaling.^87^^,^^88^

**CRITICAL:** For best results, make up fresh differentiation media no more than 3 days ahead of when it will be used. We have not rigorously tested how long differentiation media remains stable at 4°C, nor whether it can be frozen and thawed.

13. After allowing Accutase-dissociated hPSCs to recover for 16–24 h, observe cells under a microscope to evaluate whether cell density is appropriate for differentiation (Figure 1A).

***Note:*** If cells are seeded too sparsely, they will largely die when treated with differentiation media; conversely, if cells are seeded too densely, unwanted cell-types may emerge during differentiation that compromise differentiation purity (Figure 1). Evaluating the appropriateness of cell density at this stage may require some practice.

14. If cells density is appropriate, proceed by aspirating mTeSR Plus + 1 μM Thiazovivin. Then, briefly wash cells with DMEM/F12 to dilute any traces of the prior medium: add DMEM/F12, gently swirl the plate to dislodge any dead or detached cells, and then aspirate the DMEM/F12.
15. This time point is considered day 0 of differentiation. On day 0, add posterior primitive streak induction medium for 24 h.

***Note:*** 24 h of posterior primitive streak differentiation generates day-1 posterior primitive streak cells. At this stage, there should be a dramatic morphological change that can be observed under a microscope. Undifferentiated hPSCs form spiky colonies when cultured in the presence of Thiazovivin; however, during primitive streak differentiation, colonies appear larger, become more rounded, and spread out (Figure 1B).

***Note:*** There is typically some degree of cell death during primitive streak differentiation. However, excessive cell death may indicate that hPSCs were seeded too sparsely (Figure 1B). Conversely, if day-1 primitive streak colonies are too large, this may decrease differentiation purity. This is likely attributable to the uneven action of developmental signals (e.g., BMP) across an excessively-wide hPSC colony.^89^^,^^90^

16. On day 1 of differentiation, aspirate current media from plates and add fresh posterior primitive streak induction medium for an additional 24 h.

***Note:*** Washing with DMEM/F12 is unnecessary at this stage, as the posterior primitive streak medium being added is the same medium as the previous stage.

***Note:*** 48 consecutive hours of posterior primitive streak differentiation generates day 2 posterior primitive streak cells. At this stage, cells once again undergo an observable morphological change and become somewhat triangular or star-like in shape. They should also begin to spread out across the plate (Figure 1C).

17. On day 2 of differentiation, briefly wash cells with DMEM/F12, then add lateral mesoderm induction medium for 24 h.

***Note:*** 24 h of lateral mesoderm differentiation generates day-3 lateral mesoderm cells. At this stage, cells should still appear star-like in shape and form a largely confluent monolayer (Figure 1D).

***Note:*** If cells were initially seeded too densely, larger mesenchyme-like cells may start to become visible overlaying the monolayer of mesoderm cells in the culture plate (Figure 1D). This is a visual indicator of decreased differentiation purity, and should be addressed by seeding cells more sparsely (see “troubleshooting” section below).

18. On day 3 of differentiation, briefly wash cells with DMEM/F12, then add artery endothelial differentiation induction medium for 24 h.

***Note:*** 24 h of artery endothelium differentiation generates day-4 artery endothelial cells. At this stage, cells should appear more rounded in a largely confluent monolayer (Figure 1E). If cells were initially seeded too densely, swaths of mesenchyme-like cells may become more visible at this stage (Figure 1E). This is a visual indicator of decreased differentiation purity (which can be confirmed by flow cytometry for artery EC markers, such as CD144 and DLL4), and should be addressed by seeding cells more sparsely (see “troubleshooting” section below).

19. On day 3 of differentiation, coat 24-well plates with Vitronectin + DLL4-E12. Add 300 μL of Vitronectin + DLL4-E12 to each well and gently shake the plate to ensure that all wells are evenly coated. Incubate coated plates at 37°C for at least 12 h.

***Note:*** As described in later steps, day-4 artery cells will be re-seeded with high density (∼500,000 cells per cm^2^, or ∼1 million cells per well) on to Vitronectin + DLL4-E12 coated plates. When determining the number of wells to coat with Vitronectin + DLL4-E12, experimenters should consider the number of day-4 artery cells that they expect to recover. When hPSCs are seeded at optimal density, experimenters should expect to recover 1 day-4 artery cell per initially-seeded hPSC.^1^ For instance, a 10 cm dish initially seeded with 2.5 million hPSCs should yield approximately 2.5 million day-4 artery cells. In this case, the experimenter could prepare 2 wells of a 24-well plate with Vitronectin + DLL4-E12.

20. On day 4 of differentiation, retrieve pre-coated plates from the incubator and gently wash 3 times with PBS to remove any unbound DLL4-E12. Add 500 μL of hemogenic endothelium induction medium to each pre-coated well.

**CRITICAL:** As mentioned previously, soluble NOTCH ligands will actually inhibit NOTCH signaling, owing to the requirement for mechanical force to activate the NOTCH receptor.^87^^,^^88^ It is therefore critical to wash plates with PBS to ensure that any soluble NOTCH ligands are removed. Washing should be performed gently to avoid washing adhered Vitronectin + DLL4-E12 off of the well.

21. On day 4 of differentiation, briefly wash cells with DMEM/F12, and dissociate artery cells with Accutase, as described above.
  a. Aspirate DMEM/F12 and add 5 mL of Accutase to each dish. Incubate dishes at 37°C for ∼5 min or until cells begin to detach from the bottom of the dish.
  b. Transfer dissociated cells into a 50 mL conical tube with a sufficient volume of DMEM/F12 to dilute the Accutase at least 1:5. Wash dishes with DMEM/F12 to collect any remaining cells.
  c. Centrifuge the tube at 500 × g for 5 min at 4°C to pellet hPSCs.
  d. Resuspend cells in hemogenic endothelium medium for counting (described above) and seeding.
22. After cell counting is complete, adjust the volume of hemogenic endothelium medium to seed cells at 500,000 cells per cm^2^ (1 million cells per well of a 24-well plate).
  a. For example, if an experimenter counted 2 million cells, they could resuspend cells in 2 mL of hemogenic endothelium medium. Subsequently, they could dispense 1 mL of cell mixture per well of a 24-well plate. Each well should already contain 500 μL of hemogenic endothelium induction medium, as described in step 20.
  b. As described above, cells should be mixed well before seeding. Once cells have been seeded, shake the plate in a cross pattern (left, then right; up, then down) to evenly disperse cells before returning the plate to the 37°C incubator.

***Note:*** We have found that seeding cells at 500,000 cells per cm^2^ at this step reproducibly yields highly-pure hematopoietic progenitors across multiple cell lines; however, the specific density may need to be re-optimized in some cases. Typically, seeding more than 500,000 cells per cm^2^ (up to 1,000,000 cells per cm^2^) at this step does not have any negative effects on differentiation.^1^ However, seeding fewer than 400,000–500,000 cells per cm^2^ typically impairs conversion of artery cells into hemogenic endothelium,^1^ and thus decreases both the purity of differentiation and the expression of key genes, including *HLF*, in day-10 hematopoietic progenitors.^1^

23. On day 5 of differentiation, briefly wash cells with DMEM/F12 to dislodge out any dead or detached cells. At this stage, cells should be very densely packed and form a confluent monolayer (Figure 1F). Then, add hemogenic endothelium induction medium for an additional 24 h to generate day-6 hemogenic endothelium.
24. On day 6 of differentiation, aspirate current media from plates and add hemogenic endothelium induction medium for an additional 24 h. Washing with DMEM/F12 is unnecessary at this stage, as the hemogenic endothelium medium being added is the same medium as the previous stage.
25. On day 7 of differentiation, aspirate current media from plates, then add hematopoietic progenitor induction medium for 24 h. Do not wash with DMEM/F12, which may dislodge semiadherent hematopoietic progenitors (Figure 1G) emerging from the underlying EC monolayer.
26. On day 8 of differentiation, aspirate current media from plates, then add hematopoietic progenitor induction medium for an additional 24 h. Do not wash with DMEM/F12, which may dislodge semiadherent hematopoietic progenitors (Figure 1G) emerging from the underlying EC monolayer.
27. On day 9 of differentiation, do not aspirate current media from plates, as doing so may dislodge semiadherent hematopoietic progenitors (Figure 1G). Instead, add an additional working volume of hematopoietic progenitor induction medium (e.g., 1 mL) for an additional 24 h.
28. On day 10 of differentiation, collect cells for downstream assays (described below). Alternatively, cells can be frozen for later use. Methods for freezing cells have been extensively described elsewhere^40^; briefly, dissociate cells with TrypLE (Thermo Fisher Scientific, 12604013) and freeze in Cryostor CS10 (STEMCELL Technologies, 07930). Cells can be stored in liquid nitrogen for several years.

## Expected outcomes

**Figure 2:**
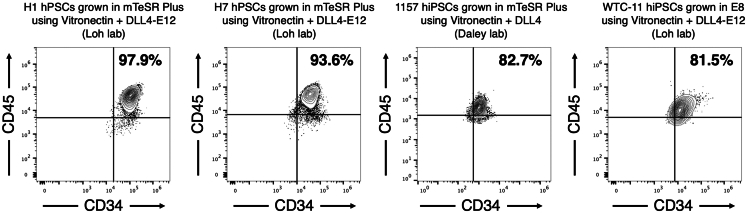
Flow cytometry of multiple hPSC lines differentiated into hematopoietic progenitors using the aforementioned protocol H1 hESCs, H7 hESCs, 1157 hiPSCs, and WTC11 hiPSCs can all be differentiated into CD34+ CD45+ hematopoietic progenitors with >80% purity.

**Figure 3:**
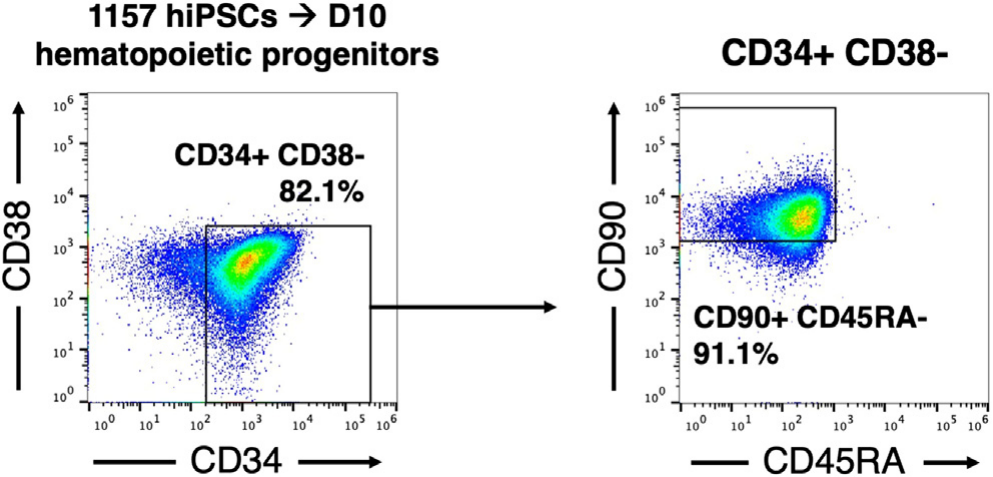
Flow cytometry of day-10 hematopoietic progenitors generated from 1157 hiPSCs 1157 hiPSCs were differentiated into CD34+ CD38- CD90+ CD45RA- hematopoietic progenitors.

This differentiation protocol typically generates CD34+ CD45+ hematopoietic progenitors with 80–100% purity (Figure 2). We assess the purity of differentiation using flow cytometry. Our flow cytometry protocol has been described elsewhere.^57^ In brief, hematopoietic progenitors are dissociated with TrypLE Express (Thermo Fisher Scientific, 12604013) and stained with anti-CD34 (BD Biosciences, 48-0349-41, used at 1:100) and anti-CD45 (BioLegend Biosciences, 304017, used at 1:100) for 30 min at 4°C, followed by analysis on a flow cytometer. Samples should be kept cold and protected from light throughout the staining process. Hematopoietic progenitors are also CD90+ (anti-CD90, BD Biosciences, 562556, used at 1:100), CD38- (anti-CD38, BD Biosciences, 563251, used at 1:100) and CD45RA- (anti-CD45RA, BioLegend, 304134, used at 1:100) (Figure 3).

**Figure 4:**
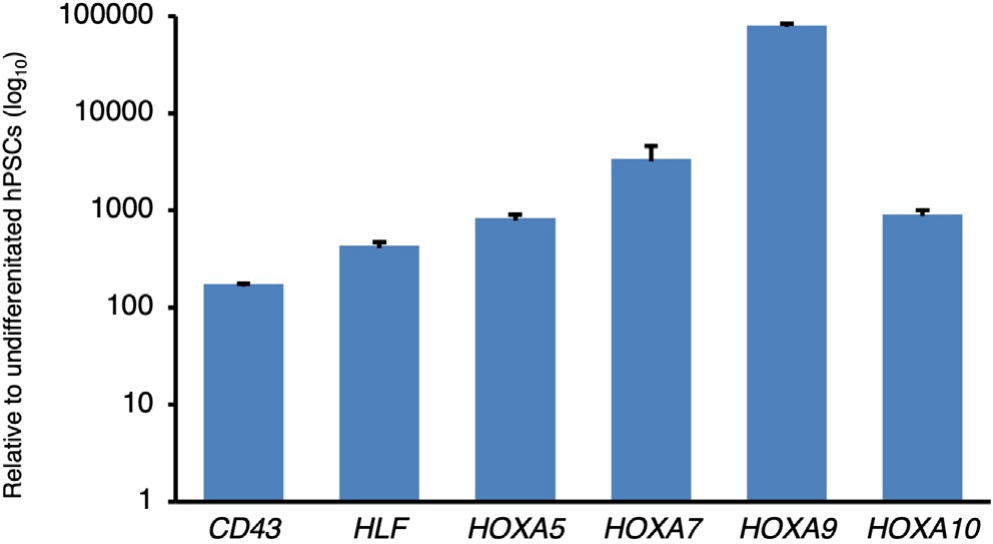
qPCR analysis of mRNA expression in day-10 hematopoietic progenitors generated from H7 hPSCs Expression is relative to undifferentiated H7 hPSCs (i.e., levels in undifferentiated hPSCs = 1.0) and is depicted in a log_10_ scale.

Quantitative PCR (qPCR) and RNA-sequencing can be used to assess the expression of HSC markers in hPSC-derived hematopoietic progenitors; these methods are described elsewhere.^1^ For instance, qPCR reveals that day-10 hematopoietic progenitors express blood markers, such as *CD43* (also known as *SPN*), which distinguishes blood cells from ECs.^91^^,^^92^ Notably, day-10 hPSC-derived hematopoietic progenitors also express *HLF* and *HOXA5-10,* which encode key HSC transcription factors^12^^,^^26^^,^^27^^,^^28^^,^^29^^,^^30^^,^^31^^,^^32^^,^^33^^,^^34^^,^^35^^,^^36^ (Figure 4).

## Limitations

This protocol to differentiate hPSCs into hematopoietic progenitors has been robustly and independently reproduced by the Loh and Daley laboratories across multiple hPSC lines (Figure 2). However, the following potential limitations should be taken into account.

First, cell seeding density is of paramount importance to achieve efficient differentiation into hematopoietic progenitors. Initial hPSC seeding density should be optimized by each individual experimenter, especially for new hPSC lines.

Second, some hPSC cell lines may differentiate less efficiently. In the authors’ collective experience, this protocol has efficiently differentiated multiple hPSC lines—including H1 hESCs, H7 hESCs, 1157 hiPSCs, and WTC11 hiPSCs—into hematopoietic progenitors (Figure 2). We expect that if cell seeding density is optimized, this differentiation protocol will yield highly-pure hematopoietic progenitors across a range of wild-type hPSC lines. Nevertheless, it remains possible that some hPSC lines may yield hematopoietic progenitors with lower purity, although we have not observed this outcome thus far.

Lastly, this protocol generates hPSC-derived *HLF*+ *HOXA*+ hematopoietic progenitors that, when transplanted into adult mice, fail to robustly engraft *in vivo*.^1^ The reasons underlying this poor engraftment remain unknown. The earliest functional HSCs that arise in the human and mouse embryo are CD144+ CD45+^93^^,^^94^, and hPSC-derived *HLF*+ *HOXA*+ hematopoietic progenitors are CD144+ CD45+^1^. Additionally, these hPSC-derived hematopoietic progenitors robustly express six signature genes (*RUNX1*, *HOXA9*, *MLLT3*, *MECOM*, *HLF*, and *SPINK2*)^1^ that demarcate the earliest hematopoietic stem and progenitor cells within the human embryo.^35^ Nevertheless, there must be crucial differences between authentic HSCs and the hPSC-derived *HLF*+ *HOXA*+ hematopoietic progenitors generated by our differentiation protocol. Recently, methods have been reported to differentiate hPSCs into hematopoietic stem and progenitor cells capable of engrafting mice,^95^^,^^96^ although whether the *in vitro* differentiated cells are fully equivalent to authentic HSCs remains to be determined.

## Troubleshooting

### Problem 1: Cell death during differentiation (related to step 13)

If executed correctly, differentiation should yield ∼1 hematopoietic progenitor per initially-seeded hPSC.^1^ While a degree of cell death should be expected during each step of differentiation (especially when cells have been seeded after being dissociated), near-complete or complete cell death during differentiation is unusual and should be addressed.

### Potential solution

• **Seeding more hPSCs.** In our experience, the most common cause for excessive cell death during differentiation is seeding too few hPSCs (Figure 1). As mentioned previously, we strongly recommend that each new experimenter should test a range of cell seeding densities for mTeSR Plus-grown hPSCs – between 40,000–50,000 hPSCs/cm^2^ (i.e., ∼2,250,000–2,750,000 hPSCs per 10 cm dish) – on day 0 of differentiation. Seeding an insufficient number of hPSCs will lead to near-complete cell death upon differentiation (Figure 1). As different hPSC lines grow at different rates, we strongly recommend that new experimenters test a range of hPSC seeding densities.
• **Avoid over-triturating hPSCs during the cell seeding process.** When seeding hPSCs, avoid over-trituration. Excessive trituration will lead to poor hPSC survival upon plating and excessive death upon differentiation.
• **Using fresh CDM2 basal medium.** CDM2 basal medium can be stored for up to 2 months at 4°C. However, prolonged storage may be deleterious, and may compromise CDM2 components like insulin that are required for cell survival. Therefore, fresh CDM2 medium should be used.
• **Ensure small molecules are completely frozen and free of precipitates.** When retrieving small molecules from the −20°C freezer, first check to ensure that the frozen aliquot is fully solid and contains no liquids. While thawing, check to ensure that no precipitates are present in the aliquot. Checking small molecules is especially important after repeated freeze-thaw cycles. The presence of liquids in the frozen aliquot or precipitates in the thawed aliquot may suggest that the quality of small molecules has been compromised, which may in turn have negative effects on cell differentiation and growth. If small molecules have precipitated, order fresh stocks or use a different aliquot prepared from the same master stock.
• **Avoid keeping cells dry for too long after aspiration.** When aspirating old differentiation media and adding either DMEM/F12 (to wash cells) or new differentiation medium, do not keep cells dry for too long, as this may negatively affect cell health.

### Problem 2: Poor differentiation efficiency (related to step 28)

We have demonstrated that multiple hPSC lines can be efficiently differentiated into hematopoietic progenitors (Figure 2). This result is reproducible across multiple independent experimenters and laboratories. If poor differentiation efficiency is encountered, attempt the following solutions.

### Potential solution

• **Seeding fewer hPSCs.** In our experience, the leading cause for poor differentiation efficiency is seeding excessive numbers of hPSCs on day 0 of differentiation. As emphasized previously, each new experimenter should test a range of cell seeding densities for mTeSR Plus-grown hPSCs on day 0 of differentiation. Generally speaking, seeding too many hPSCs will lead to poor differentiation efficiency, possibly due to uneven action of developmental signals (e.g., BMP) across an excessively-wide hPSC colony.^89^^,^^90^ If hPSCs are seeded too densely, cells may become overcrowded and the presence of contaminating cell-types may become visually apparent as early as day 2 of differentiation (Figure 1).
• **Use of CDM2 and CDM3 basal media.** Differentiation should be conducted with the CDM2 and CDM3 basal media described here. Do not substitute CDM2 or CDM3 with different basal media.
• **Double check small molecule and growth factor reconstitution and storage.** Resuspend lyophilized small molecules and growth factors only with the diluents and at the concentrations suggested above. Improper reconstitution of differentiation factors can compromise stability of factors, and resultantly impair differentiation.
• **Double check small molecule identity.** Use the exact small molecules specified above. Do not substitute them for alternative substitutes. Each small molecule has its own set of potential “off-target” effects. For example, replacing (1) the BMP pathway inhibitor DMH1 with the alternative BMP pathway inhibitor LDN193189 or (2) the TGF-β pathway inhibitor SB505124 with the alternative TGF-β pathway inhibitor A8301 will compromise differentiation.

## Resource availability

### Lead contact

Further information and requests for resources and reagents should be directed to and will be fulfilled by the lead contact, Kyle M. Loh (kyleloh@stanford.edu).

### Technical contact

Technical questions on executing this protocol should be directed to and will be fulfilled by the technical contact, Sherry Li Zheng (slzheng@stanford.edu).

### Materials availability

The above protocol does not entail the use of any new, unique reagents.

### Data and code availability

The above protocol does not entail the use of any new datasets or codes.

## Acknowledgments

We thank Liying Ou, Aaron McCarty, Catherine Carswell-Crumpton, Mike Alvarez, Laura Dunkin-Hubby, Linda Heneghan, the Stanford Institute for Stem Cell Biology & Regenerative Medicine, and the Stanford Diabetes Research Center (NIH P30DK116074) for infrastructure support. This work was supported by NIH Director’s Early Independence Award DP5OD024558 (K.M.L.), the Breakthrough T1D Northern California Center of Excellence (K.M.L.), the Stanford Ludwig Center for Cancer Stem Cell Research and Medicine (K.M.L.), the Stanford Beckman Center for Molecular and Genetic Medicine (K.M.L.), the Bill and Melinda Gates Foundation (INV-034912; K.M.L.), and the Anonymous, Fickel, and Gilbert families (K.M.L.). S.L.Z. was supported by NSF and Stanford Graduate Fellowships and NIH T32GM007790. J.L.F. was supported by NDSEG and Stanford Bio-X Fellowships. J.Y.C. was supported by the Goldwater Scholarship, New Science Research Fellowship, and Time Initiative Fellowship. A.T.N. and A.C. were supported by the California Institute for Regenerative Medicine Bridges Program (TB1-01195). L.T.A. is a Siebel Investigator and Additional Ventures Catalyst to Independence Fellow. K.M.L. is a Human Frontier Science Program Young Investigator (RGY0069/2019), Packard Foundation Fellow, Pew Scholar, Baxter Foundation Faculty Scholar, and The Anthony DiGenova Endowed Faculty Scholar.

## Author contributions

J.L.F., A.T.N., A.C., L.T.A., and K.M.L. developed the method to differentiate hPSCs into hematopoietic progenitors. S.L.Z., J.Y.C., C.L., E.L., and G.Q.D. tested the robustness and reproducibility of the hPSC differentiation method. S.L.Z. and K.M.L. drafted the manuscript, which was edited with input from all authors.

## Declaration of interests

Stanford University has filed patent applications related to blood and immune cell differentiation, with K.M.L. and J.L.F. listed as inventors. J.L.F., A.T.N., and A.C. are respectively affiliated with Azalea Therapeutics, University of California Davis, and Orca Bio but contributed to this work when affiliated with Stanford University. G.Q.D. is an inventor on intellectual property filed by Boston Children’s Hospital pertaining to derivation of blood lineages from pluripotent stem cells.
